# Supplementary material for: The Systems Biology Research Tool: evolvable open-source software
Source: BMC Syst Biol. 2008 Jun 29;2:55. doi: 10.1186/1752-0509-2-55 (PMC2446383; doi:10.1186/1752-0509-2-55)
Supplement: Additional file 1 — SBRT Archive. An archive of the current version of the Systems Biology Research Tool. [file 1752-0509-2-55-S1.zip › sbrt-1.4.0/doc/developers_guide/api/sbrt/kernel/fba/comp/CdHarFluxVectorComputer.html]

CdHarFluxVectorComputer


|  |  |  |
| --- | --- | --- |
| **Overview** \| **Package** \| **Class** \| **Use** \| **Tree** \| **Deprecated** \| **Index** \| **Help** | |  |
| **PREV CLASS**   **NEXT CLASS** | **FRAMES**    **NO FRAMES**     **All Classes** |  |
| SUMMARY: NESTED \| FIELD \| CONSTR \| METHOD | DETAIL: FIELD \| CONSTR \| METHOD |  |


---


## sbrt.kernel.fba.comp Class CdHarFluxVectorComputer

```
java.lang.Object
  sbrt.kernel.fba.comp.AbstractCvComputer
      sbrt.kernel.fba.comp.CdHarFluxVectorComputer
```

**All Implemented Interfaces:**: FluxCvComputer

---

``` public class CdHarFluxVectorComputer extends AbstractCvComputer ```

This class is used to compute randomly distributed vectors within flux space.
It uses a coordinate-direction hit-and-run algorithm.

**Author:**
:   This class was written and documented by Jeremiah Wright while in the
    Wagner lab.

---

| **Nested Class Summary** | |
| --- | --- |
| `class` | `CdHarFluxVectorComputer.Results`             This class is used to represent the results of a coordinate-direction hit-and-run flux vector computation. |


| **Field Summary** | |
| --- | --- |
| `static double` | `DEFAULT_TOLERANCE`             The default constraint tolerance. |


**Constructor Summary**

`CdHarFluxVectorComputer(IrrevRxnFluxome fluxome,
LinearSystemSolution<java.lang.String> systemSolution)`
  
          Constructs a new random flux vector computer for the provided fluxome.


| **Method Summary** | |
| --- | --- |
| `double` | `getConstraintTolerance()`             Returns the constraint tolerance. |
| `Interval` | `getFluxConstraint(java.lang.String rxnName)`             Returns the current flux constraint on the reaction with the provided name. |
| `java.util.Map<java.lang.String,Interval>` | `getFluxConstraints()`             Returns all current flux constraints. |
| `double` | `getMinChordLength()`             Returns the minimum length a computed chord must have to be considered successful. |
| `Interval` | `getRxnExprConstraint(MathExpr<java.lang.String> rxnNameExp)`             Returns the current constraint on the provided expression. |
| `java.util.Map<LinearComb<java.lang.String>,Interval>` | `getRxnExprConstraints()`             Returns all current flux expression constraints. |
| `void` | `restoreFluxConstraint(java.lang.String reactionName)`             Restores the flux constraint for the reaction with the provided name to the value contained in the fluxome. |
| `void` | `restoreRxnExprConstraint(LinearComb<java.lang.String> expr)`             Restores the constraint on the provided flux expression to the value contained in the fluxome. |
| `void` | `restoreRxnExprConstraint(MathExpr<java.lang.String> expr)`             Restores the constraint on the provided flux expression to the value contained in the fluxome. |
| `void` | `restoreRxnExprConstraints()`             Restores the constraints on all flux expressions to the values contained in the fluxome. |
| `CdHarFluxVectorComputer.Results` | `run()`             Computes the next random flux vector from within the interior of the convex polytope defined by the current set of constraints. |
| `void` | `setConstraintTolerance(double tolerance)`             Sets the constraint tolerance. |
| `void` | `setFluxConstraint(java.lang.String reactionName, Interval c)`             Changes the flux constraint of the reaction with the provided name. |
| `void` | `setInitialFluxVector(java.util.Map<java.lang.String,java.lang.Double> vector)`             Sets the initial flux vector to the one provided. |
| `void` | `setMinChordLength(double minChordLength)`             Sets the minimum chord length. |
| `void` | `setPointsPerPoint(int pointsPerPoint)`             Sets the number of points to generate for each point added to the specified results manager. |
| `void` | `setRandom(java.util.Random random)`             Sets the random number generator. |
| `void` | `setRxnExprConstraint(LinearComb<java.lang.String> rxnExpr, Interval c)`             Changes the constraint on the provided linear combination of fluxes. |
| `void` | `setRxnExprConstraint(MathExpr<java.lang.String> rxnExpr, Interval c)`             Changes the constraint on the provided mathematical expression of fluxes. |

**Methods inherited from class sbrt.kernel.fba.comp.AbstractCvComputer**

`checkFluxConstraint, checkNumber, checkRxnExpr, checkRxnExprConstraint, checkRxnName, checkRxnNames, getFluxome, getRxnExpr, getRxnExpr, restoreFluxConstraints, restoreFluxConstraints, restoreRxnExprConstraints, setFluxConstraints, setRxnExprConstraints`

**Methods inherited from class java.lang.Object**

`clone, equals, finalize, getClass, hashCode, notify, notifyAll, toString, wait, wait, wait`

**Field Detail**

### DEFAULT\_TOLERANCE

```
public static final double DEFAULT_TOLERANCE
```

:   The default constraint tolerance.

    **See Also:**: Constant Field Values


**Constructor Detail**

### CdHarFluxVectorComputer

```
public CdHarFluxVectorComputer(IrrevRxnFluxome fluxome,
                               LinearSystemSolution<java.lang.String> systemSolution)
```

:   Constructs a new random flux vector computer for the provided fluxome.

    **Parameters:**: `fluxome` - the fluxome for which random flux vectors will be computed.: `systemSolution` - a solution to the system of linear equations formed by the provided fluxome.


**Method Detail**

### setRandom

```
public void setRandom(java.util.Random random)
```

:   Sets the random number generator.

    :   **Parameters:**: `random` - the random number generator with which all psuedorandom numbers are to be produced.

---


### setPointsPerPoint

```
public void setPointsPerPoint(int pointsPerPoint)
```

:   Sets the number of points to generate for each point added to the
    specified results manager.

    :   **Parameters:**: `pointsPerPoint` - the number of points to generate for each point added to the specified results manager.

---


### setConstraintTolerance

```
public void setConstraintTolerance(double tolerance)
```

:   Sets the constraint tolerance. This is the amount by which constraints
    can be violated.

    :   **Parameters:**: `tolerance` - the amount by which constaints can be violated. This should be a small positive number, like 1E-9 for example.

---


### setMinChordLength

```
public void setMinChordLength(double minChordLength)
```

:   Sets the minimum chord length. If a computed chord is shorter than the
    provided amount, the move is considered wasted. This can happen if the
    random walker gets stuck in a corner.

    :   **Parameters:**: `minChordLength` - the minimum length a chord must have to be considered valid. This should be a small positive number, like 1E-9 for example.

---


### getConstraintTolerance

```
public double getConstraintTolerance()
```

:   Returns the constraint tolerance. This is the amount by which constraints
    can be violated.

    :   **Returns:**: the constraint tolerance. **See Also:**: `setConstraintTolerance(double)`

---


### getMinChordLength

```
public double getMinChordLength()
```

:   Returns the minimum length a computed chord must have to be considered
    successful. Chords with a length smaller than the returned value are
    considered wasted moves.

    :   **Returns:**: the minimum chord length. **See Also:**: `setMinChordLength(double)`

---


### run

```
public CdHarFluxVectorComputer.Results run()
```

:   Computes the next random flux vector from within the interior of the
    convex polytope defined by the current set of constraints.

    :   **Returns:**: the results of computing the next random flux vector.

---


### setInitialFluxVector

```
public void setInitialFluxVector(java.util.Map<java.lang.String,java.lang.Double> vector)
```

:   Sets the initial flux vector to the one provided.

    :   **Parameters:**: `vector` - reaction names mapped to the flux of their corresponding reaction.

---


### setFluxConstraint

```
public void setFluxConstraint(java.lang.String reactionName,
                              Interval c)
```

:   Changes the flux constraint of the reaction with the provided name.

    :   **Parameters:**: `reactionName` - the name of the reaction whose flux constraint is to be changed.: `c` - the new constraint.

---


### restoreFluxConstraint

```
public void restoreFluxConstraint(java.lang.String reactionName)
```

:   Restores the flux constraint for the reaction with the provided name to
    the value contained in the fluxome.

    :   **Parameters:**: `reactionName` - the name of the reaction whose flux constraint is to be restored.

---


### setRxnExprConstraint

```
public void setRxnExprConstraint(MathExpr<java.lang.String> rxnExpr,
                                 Interval c)
```

:   Changes the constraint on the provided mathematical expression of fluxes.

    :   **Parameters:**: `rxnExpr` - a `LinearComb` of reaction names.: `c` - the new constraint.

---


### setRxnExprConstraint

```
public void setRxnExprConstraint(LinearComb<java.lang.String> rxnExpr,
                                 Interval c)
```

:   Changes the constraint on the provided linear combination of fluxes.

    :   **Parameters:**: `rxnExpr` - a linear combination of reaction names.: `c` - the new constraint.

---


### restoreRxnExprConstraint

```
public void restoreRxnExprConstraint(MathExpr<java.lang.String> expr)
```

:   Restores the constraint on the provided flux expression to the value
    contained in the fluxome.

    :   **Parameters:**: `expr` - the `LinearComb` of reaction names whose contraint is to be restored.

---


### restoreRxnExprConstraint

```
public void restoreRxnExprConstraint(LinearComb<java.lang.String> expr)
```

:   Restores the constraint on the provided flux expression to the value
    contained in the fluxome.

    :   **Parameters:**: `expr` - the linear combination of reaction names whose contraint is to be restored.

---


### restoreRxnExprConstraints

```
public void restoreRxnExprConstraints()
```

:   Restores the constraints on all flux expressions to the values contained
    in the fluxome.

---


### getFluxConstraints

```
public java.util.Map<java.lang.String,Interval> getFluxConstraints()
```

:   Returns all current flux constraints.

    :   **Returns:**: reaction names mapped to their respective constraint.

---


### getRxnExprConstraints

```
public java.util.Map<LinearComb<java.lang.String>,Interval> getRxnExprConstraints()
```

:   Returns all current flux expression constraints.

    :   **Returns:**: mathematical expressions of reaction names mapped to their respective constraint.

---


### getRxnExprConstraint

```
public Interval getRxnExprConstraint(MathExpr<java.lang.String> rxnNameExp)
```

:   Returns the current constraint on the provided expression.

    :   **Parameters:**: `rxnNameExp` - the mathematical expression of reaction names whose constraint is to be returned. **Returns:**: the current constraint on the provided expression.

---


### getFluxConstraint

```
public Interval getFluxConstraint(java.lang.String rxnName)
```

:   Returns the current flux constraint on the reaction with the provided
    name.

    :   **Parameters:**: `rxnName` - the name of the reaction whose flux constraint is to be returned. **Returns:**: the current flux constraint on the reaction with the provided name.


---


|  |  |  |
| --- | --- | --- |
| **Overview** \| **Package** \| **Class** \| **Use** \| **Tree** \| **Deprecated** \| **Index** \| **Help** | |  |
| **PREV CLASS**   **NEXT CLASS** | **FRAMES**    **NO FRAMES**     **All Classes** |  |
| SUMMARY: NESTED \| FIELD \| CONSTR \| METHOD | DETAIL: FIELD \| CONSTR \| METHOD |  |


---
